# Supplementary material for: Quorum sensing controls Vibrio cholerae multicellular aggregate formation
Source: eLife. 2018 Dec 24;7:e42057. doi: 10.7554/eLife.42057 (PMC6351105; doi:10.7554/eLife.42057)
Supplement: Supplementary file 1. [file elife-42057-supp1.docx]

**Supplementary Table 1: Strain list**

| **Strain or plasmid** | **Relevant features** | **Reference** |
| --- | --- | --- |
| ***V. cholerae*** |  |  |
| C6706str2 | Wild type, El Tor *V. cholerae* | (Thelin and Taylor, 1996) |
| BB-Vc0184 | *luxO* D61A | S. Svenningsen, unpublished |
| BB-Vc0201 | *luxO* D61E | (Waters et al., 2008) |
| BB-Vc0093 | Wild type, El Tor *V. cholerae* lacZ:P*_tac_*-mKO:lacZ | J. Yan, unpublished |
| BB-Vc0208 | Δ*vpsL luxO* D61A | This study |
| BB-Vc0209 | Δ*vpsL luxO* D61E | This study |
| BB-Vc0206 | *luxO* D61A lacZ:P*_tac_*-mKO:*l*acZ | This study |
| BB-Vc0204 | *luxO* D61E lacZ:P*_tac_*-mKO:*l*acZ | This study |
| BB-Vc0100 | vpvC W240R lacZ:P*_tac_*-mKO:*lacZ* | (Yan et al., 2016) |
| BB-Vc0096 | Δ*vpsL* lacZ:P*_tac_*-mKO:*lacZ* | (Yan et al., 2017) |
| BB-Vc0211 | Δ*vpsL luxO* D61A lacZ:P*_tac_*-mKO:lacZ | This study |
| BB-Vc0212 | Δ*vpsL* *luxO* D61A lacZ:P*_tac_*-mKate2:lacZ | This study |
| BB-Vc0210 | Δ*vpsL* *luxO* D61A lacZ:P*_tac_*-mTFP1:lacZ | This study |
| BB-Vc0214 | Δ*vpsL luxO* D61E lacZ:P*_tac_*-mKO:lacZ | This study |
| BB-VC0232 | Δ*vpsL* Δ*vpsN luxO* D61A lacZ:P*_tac_*-mKO:lacZ | This study |
| BB-VC0233 | Δ*vpsL* Δ*asnB* *luxO* D61A lacZ:P*_tac_*-mKO:lacZ | This study |
| BB-Vc0286 | Δ*vpsL* Δ*carR luxO* D61A lacZ:P*_tac_*-mKO:lacZ | This study |
| BB-Vc0287 | Δ*vpsL* Δ*tcpA* *luxO* D61A lacZ:P*_tac_*-mKO:lacZ | This study |
| BB-Vc0288 | Δ*vpsL* Δ*pilA* *luxO* D61A lacZ:P*_tac_*-mKO:lacZ | This study |
| BB-Vc0289 | Δ*vpsL* Δ*mshA* *luxO* D61A lacZ:P*_tac_*-mKO:lacZ | This study |
| BB-Vc0290 | Δ*cqsA* Δ*luxQ* Δ*vpsS* Δ*cqsR* Δ*vpsL* lacZ:P*_tac_*-mKO:lacZ | This study |
| BB-Vc0291 | Δ*luxS* Δ*cqsS* Δ*vpsS* Δ*cqsR* Δ*vpsL lacZ*:P*_tac_*-mKO:*lacZ* | This study |
| BB-VC0292 | Δ*cqsA* Δ*luxS* *lacZ*:*P*_tac_*-mKO*:*lacZ* | This study |
| BB-VC0293 | Δ*cqsA* Δ*luxQ* Δ*vpsS* Δ*cqsR* Δ*vpsL* pBB1 | This study |
| BB-Vc0230 | Δ*vpsL* Δ*aphA* *luxO* D61A lacZ:P*_tac_*-mKO:lacZ | This study |
| BB-Vc0231 | Δ*vpsL* Δ*hapR* *luxO* D61A lacZ:P*_tac_*-mKO:lacZ | This study |
| BB-Vc0234 | Δ*vpsL* Δ*aphA* Δ*hapR* *luxO* D61A lacZ:P*_tac_*-mKO:lacZ | This study |
| BB-Vc0228 | Δ*vpsL* Δ*aphA* *luxO* D61E lacZ:P*_tac_*-mKO:lacZ | This study |
| BB-Vc0229 | Δ*vpsL* Δ*hapR* *luxO* D61E lacZ:P*_tac_*-mKO:lacZ | This study |
| BB-Vc0235 | Δ*vpsL* Δ*aphA* Δ*hapR* *luxO* D61E lacZ:P*_tac_*-mKO:lacZ | This study |
| BB-Vc0294 | Δ*vpsL* Δ*dns* *luxO* D61A lacZ:P*_tac_*-mKO:lacZ | This study |
| BB-Vc0295 | Δ*vpsL* Δ*xds* *luxO* D61A lacZ:P_tac_-mKO:lacZ | This study |
| BB-Vc0296 | Δ*vpsL* Δ*xds* Δ*dns* *luxO* D61A lacZ*:P_tac_*-mKO:lacZ | This study |
| BB-Vc0308 | Δ*vpsL* Δ*xds* Δ*dns* *luxO* D61A | This study |
| BB-Vc0297 | Δ*vpsL* Δ*dns* *luxO* D61E lacZ:P*_tac_*-mKO:lacZ | This study |
| BB-Vc0298 | Δ*vpsL* Δ*xds* *luxO* D61E lacZ:P*_tac_*-mKO:lacZ | This study |
| BB-Vc0299 | Δ*vpsL* Δ*xds* Δ*dns* *luxO* D61E lacZ:P*_tac_*-mKO:lacZ | This study |
| BB-VC0309 | Δ*vpsL* Δ*xds* Δ*dns* *luxO* D61E | This study |
| BB-Vc0310 | Δ*vpsL luxO* D61A *lacZ*:*P_hapR_*-*hapR*:*IacZ* | This study |
| BB-Vc0219 | Δ*vpsL* Δ*hapR* *luxO* D61A | This study |
| BB-Vc0313 | Δ*vpsL* Δ*vc0175*:*Kan^R^ luxO* D61A | This study |
| BB-Vc0314 | Δ*vpsL* Δ*vc0176*:*Kan^R^ luxO* D61A | This study |
| BB-Vc0315 | Δ*vpsL* Δ*vc1904*:*Kan^R^* (*lrp*) *luxO* D61A | This study |
| BB-Vc0316 | Δ*vpsL* Δ*vc0487*:*Kan^R^* (*glmS*) *luxO* D61A | This study |
| BB-Vc0317 | Δ*vpsL* Δ*vc2199*:*Kan^R^* (*flgC*) *luxO* D61A | This study |
| BB-Vc0318 | Δ*vpsL* Δ*vc2562*:*Kan^R^* (*cpdB*) *luxO* D61A | This study |
| BB-Vc0319 | Δ*vpsL* Δ*vc1836*:*Kan^R^* (*tolB*) *luxO* D61A | This study |
| BB-Vc0320 | Δ*vpsL* Δ*vc0647*:*Kan^R^* (*pnp*) *luxO* D61A | This study |
| BB-Vc0321 | Δ*vpsL* Δ*vc2091*:*Kan^R^* (*sdhC*) *luxO* D61A | This study |
| BB-Vc0322 | Δ*vpsL* Δ*vc0092*:*Kan^R^* (*lexA*) *luxO* D61A | This study |
| BB-Vc0323 | Δ*vpsL* Δ*vc2453* (*varS*) *luxO* D61A | This study |
| BB-Vc0324 | Δ*vpsL* Δ*vc0576* (*sspA*) *luxO* D61A | This study |
| BB-Vc0311 | Δ*vpsL* Δ*hapR* *luxO* D61A *lacZ*:*P_hapR_*-*hapR*:*IacZ* | This study |
| BB-Vc0312 | Δ*vpsL* Δ*xds* Δ*dns* *luxO* D61A *vc1807*:*Kan^R^* | This study |
| MJ413 | Δ*vpsL* *luxO* D61A *vc1807*:*Kan^R^* | This study |
| MJ420 | *ΔvpsL Δxds* *Δdns luxO D61A lacZ:P_dns_-dns:IacZ vc1807:Kan^R^* | This study |
|  |  |  |
| **Plasmids** |  |  |
| BB-Ec0199 | pKAS32 Suicide vector, Amp^R^ Sm^S^ | (Skorupski and Taylor, 1996) |
| BB-Ec0363 | pKAS32 Δ*vpsL* | (Nadell and Bassler, 2011) |
| BB-Ec0360 | pKAS32 lacZ:P_tac_-mTFP1:lacZ | (Nadell et al., 2015) |
| BB-Ec0361 | pKAS32 lacZ:P_tac_-mKate2:lacZ | (Nadell et al., 2015) |
| BB-Ec0362 | pKAS32 lacZ:P_tac_-mKO:lacZ | (Nadell et al., 2015) |
| BB-Ec0743 | pKAS32 Δ*cqsA* | (Miller et al., 2002) |
| BB-Ec0742 | pKAS32 Δ*luxS* | (Miller et al., 2002) |
| BB-Ec0744 | pKAS32 Δ*vpsN* (*vc0936*) | This study |
| BB-Ec0745 | pKAS32 Δ*asnB* (*vc0991*) | This study |
| BB-Ec0746 | pKAS32 Δ*carR* (*vc1320*) | This study |
| BB-Ec0747 | pKAS32 Δ*tcpA* | This study |
| BB-Ec0748 | pKAS32 Δ*pilA* | This study |
| BB-Ec0749 | pKAS32 Δ*mshA* | This study |
| BB-Ec0591 | pKAS32 Δ*hapR* | (Zhu et al., 2002) |
| BB-Ec0595 | pKAS32 Δ*aphA* | (Rutherford et al., 2011) |
| BB-Ec0610 | pKAS32 Δ*dns* | J. Yan, unpublished |
| BB-Ec0611 | pKAS32 Δ*xds* | J. Yan, unpublished |
| BB-Ec0750 | pKAS Δ*varS* | (Lenz et al., 2005) |
| BB-Ec0751 | pKAS Δ*sspA* (*vc0576*) | M. Miller, unpublished |
| BB-Ec0752 | pKAS32 *lacZ*:P_hapR_-*hapR*:*lacZ* | B. Hammer, unpublished |
| BB-Ec0224 | cosmid carrying *V. harveyi luxCDABE* operon | (Miller et al., 2002) |
| BB-Ec0348 | pRL27C Tn5 delivery vector, Kan^R^ | (Larsen et al., 2002) |

**Bibliography**

Antonova, E.S., Hammer, B.K., 2011. Quorum-sensing autoinducer molecules produced by members of a multispecies biofilm promote horizontal gene transfer to *Vibrio cholerae*. FEMS Microbiology Letters 322, 68–76. https://doi.org/10.1111/j.1574-6968.2011.02328.x

Lenz, D.H., Miller, M.B., Zhu, J., Kulkarni, R.V., Bassler, B.L., 2005. CsrA and three redundant small RNAs regulate quorum sensing in *Vibrio cholerae*. Molecular Microbiology 58, 1186–1202. https://doi.org/10.1111/j.1365-2958.2005.04902.x

Miller, M.B., Skorupski, K., Lenz, D.H., Taylor, R.K., Bassler, B.L., 2002. Parallel Quorum Sensing Systems Converge to Regulate Virulence in *Vibrio cholerae*. Cell 110, 303–314. https://doi.org/10.1016/S0092-8674(02)00829-2

Nadell, C.D., Drescher, K., Wingreen, N.S., Bassler, B.L., 2015. Extracellular matrix structure governs invasion resistance in bacterial biofilms. ISME J 9, 1700–1709. https://doi.org/10.1038/ismej.2014.246

Thelin, K.H., Taylor, R.K., 1996. Toxin-coregulated pilus, but not mannose-sensitive hemagglutinin, is required for colonization by *Vibrio cholerae* O1 El Tor biotype and O139 strains. Infect. Immun. 64, 2853–2856.

Waters, C.M., Lu, W., Rabinowitz, J.D., Bassler, B.L., 2008. Quorum Sensing Controls Biofilm Formation in *Vibrio cholerae* through Modulation of Cyclic Di-GMP Levels and Repression of vpsT. Journal of Bacteriology 190, 2527–2536. https://doi.org/10.1128/JB.01756-07

Yan, J., Nadell, C.D., Bassler, B.L., 2017. Environmental fluctuation governs selection for plasticity in biofilm production. The ISME Journal 11, 1569–1577. https://doi.org/10.1038/ismej.2017.33

Yan, J., Sharo, A.G., Stone, H.A., Wingreen, N.S., Bassler, B.L., 2016. *Vibrio cholerae* biofilm growth program and architecture revealed by single-cell live imaging. PNAS. 113, E5337-5343. https://doi.org/10.1073/pnas.1611494113

Zhu, J., Miller, M.B., Vance, R.E., Dziejman, M., Bassler, B.L., Mekalanos, J.J., 2002. Quorum-sensing regulators control virulence gene expression in *Vibrio cholerae*. PNAS 99, 3129–3134. https://doi.org/10.1073/pnas.052694299
