# Supplementary material for: Quorum sensing controls Vibrio cholerae multicellular aggregate formation
Source: eLife. 2018 Dec 24;7:e42057. doi: 10.7554/eLife.42057 (PMC6351105; doi:10.7554/eLife.42057)
Supplement: Supplementary file 2. [file elife-42057-supp2.docx]

**Supplementary Table 2: Primer list**

| **Primer description** | **Direction** | **5’→3’ sequence (cut sites are underlined)** |
| --- | --- | --- |
| *ΔvpsN, pKAS, AvrII* | fwd | AAACCTAGGGGCCTTCTCGGTGATTGAATCG |
| *ΔvpsN, pKAS* | rev | TCTTGTGTACCGTTACACCCTAAAAGGGTTACATCACGCCCTGAAC |
| *ΔvpsN, pKAS* | fwd | GTCTGTTCAGGGCGTGATGTAACCCTTTTAGGGTGTAACGGTACACAAGATGACAG |
| *ΔvpsN, pKAS, KpnI* | rev | AAAGGTACCGGGCCGTAACGCTTAGAAAACTC |
| *ΔvpsN* | fwd | ACCGACTCTGATGGCGATGG |
| *ΔasnB, pKAS, AvrII* | fwd | AAACCTAGGGGGCTTTCTGCTCACCCG |
| *ΔasnB, pKAS* | rev | GCGCTGCTGGCTAGAACTTCAAGTCATGAATTCCTTTTAAATGTTTTTTAATCTGTTGGACTTAAT |
| *ΔasnB, pKAS* | fwd | TAAAAAACATTTAAAAGGAATTCATGACTTGAAGTTCTAGCCAGCAGC |
| *ΔasnB, pKAS, KpnI* | rev | AAAGGTACCGCAATACTGGTTTTCTCAGAATTCAGTTG |
| *ΔasnB* | fwd | GCATTCAACATTTACAGTGAAGCGGG |
| *ΔcarR, pKAS, AvrII* | fwd | AAACCTAGGGCCCATCCAAATCGAGCG |
| *ΔcarR, pKAS* | rev | AGGTTTCCTTTAAATCAACTTAGGGGGGACCTCGTATTTACGGTTTATC |
| *ΔcarR, pKAS* | fwd | GATAAACCGTAAATACGAGGTCCCCCCTAAGTTGATTTAAAGGAAACCTATGCGC |
| *ΔcarR, pKAS, KpnI* | rev | AAAGGTACCGCCCTTGTGAGCAATTGAGGG |
| *ΔcarR, pKAS* | fwd | GAGAAGTTACTGAGTTTAGCGAACAGC |
| Δ*tcpA*, pKAS | fwd | GGCCAATGGTATCAACATGAATTAGC |
| Δ*tcpA*, pKAS | rev | ATTATTAAATTATCAAGATAATGGTGTAGAATTTATATAACTCCACCATTTGTGTTTTA |
| Δ*tcpA*, pKAS | fwd | TAAAACACAAATGGTGGAGTTATATAAATTCTACACCATTATCTTGATAATTTAATAAT |
| Δ*tcpA*, pKAS | rev | CCTTAGCACTCTCTGAACTAAAGTTTGC |
| Δ*tcpA*, pKAS, KpnI | fwd | AGGAGGGGTACCGATTTAGCAAGGTTACCGGGG |
| Δ*tcpA*, pKAS, AvrII | rev | AGGAGGCCTAGGTGTCCAGCAGTTGAAAATGAACTCACC |
| Δ*tcpA*, pKAS | fwd | GACTGCTGTCCGTGTCGCA |
| Δ*tcpA*, pKAS | rev | CGTTGAATTTCCGGGTTGGAAGG |
| Δ*mshA*, pKAS | fwd | GTTCTAGAGGTACCGGTTGTTAACGGGCGGTCGATGTTCAGTGATATGCT |
| Δ*mshA*, pKAS | rev | GTTACATGCACGAGCCATATTTAAACTCTCTTTCATGTGAATACGCAGCA |
| Δ*mshA*, pKAS | fwd | TGCTGCGTATTCACATGAAAGAGAGTTTAAATATGGCTCGTGCATGTAAC |
| Δ*mshA*, pKAS | rev | CGCCATATGCATCCTAGGCCTATTGTTTGTAGTGGGTTCGCTCTGTAGC |
| Δ*mshA*, pKAS | fwd | GCTACAGAGCGAACCCACTACAAACAATAGGCCTAGGATGCATATGGCG |
| Δ*mshA*, pKAS | rev | CCGCCAGCTACAAGTCACTATACGAACGTTAACAACCGGTACCTCTAGAAC |
| Δ*pilA*, pKAS | fwd | GTTCTAGAGGTACCGGTTGTTAACGTTCCGCAGCGTGATCGACACTCTAAAG |
| Δ*pilA*, pKAS | rev | GATAGCAACAAGGTTGGTGAGCAATGCCTTGCTACACAAGGGGGGAA |
| Δ*pilA*, pKAS | fwd | TTCCCCCCTTGTGTAGCAAGGCATTGCTCACCAACCTTGTTGCTATC |
| Δ*pilA*, pKAS | rev | CGCCATATGCATCCTAGGCCTATTTGGGGCCGGTCATTAAAATCATTCC |
| Δ*pilA*, pKAS | fwd | GGAATGATTTTAATGACCGGCCCCAAATAGGCCTAGGATGCATATGGCG |
| Δ*pilA*, pKAS | rev | CTTTAGAGTGTCGATCACGCTGCGGAACGTTAACAACCGGTACCTCTAGAAC |
| *Δvc0092::KanR (lexA)* | fwd | GTTTAGCGCTTGGCACCAGT |
| *Δvc0092::KanR (lexA)* | rev | ATTCCGGGGATCCGTCGACCATGAGTCACCTGTCTTTTTATACAGTTGAC |
| *Δvc0092:KanR (lexA)* | fwd | CTGTATAAAAAGACAGGTGACTCATGGTCGACGGATCCCCGGAAT |
| *Δvc0092:KanR (lexA)* | rev | TTGAGAATTTATCTCAACAATCTTCATGTAGGCTGGAGCTGCTTC |
| *Δvc0092:KanR (lexA)* | fwd | GAAGCAGCTCCAGCCTACATGAAGATTGTTGAGATAAATTCTCAACAACTTGC |
| *Δvc0092:KanR (lexA)* | rev | CAACCCTCGATATCGATCAAGTGACT |
| *Δvc0092:KanR (lexA)* | fwd | GCATTTCCGAGTGAAATGGGAGG |
| *Δvc0092:KanR (lexA)* | rev | GATCATAGACCAAGCCATTATCGACCAAG |
| *Δvc0175:KanR* | fwd | GCAAAGTACGAATCTAAGTCTACACAGTAATGAG |
| *Δvc0175:KanR* | rev | ATTCCGGGGATCCGTCGACCAACCTGAAATTATGAAACTTATTTCTATACTCTCAGGT |
| *Δvc0175:KanR* | fwd | GAAATAAGTTTCATAATTTCAGGTTGGTCGACGGATCCCCGGAAT |
| *Δvc0175:KanR* | rev | AAGCTAGTCTTGGATGCTCTCTTCTTCTGTAGGCTGGAGCTGCTTC |
| *Δvc0175:KanR* | fwd | GAAGCAGCTCCAGCCTACAGAAGAAGAGAGCATCCAAGACTAGCT |
| *Δvc0175:KanR* | rev | GATCATCATAGTGTTTTCAATCCCGCT |
| *Δvc0175:KanR* | fwd | CAACATATAGTATTTCACTCATCTTTCATACCACCC |
| *Δvc0175:KanR* | rev | GTGCAATTACCGTATTGGGGAGAAG |
| *Δvc0176:KanR* | fwd | TCATCACGAGCATCATCATCCATCTC |
| *Δvc0176:KanR* | rev | GGCGACCTTTGAGCAAGCATTC |
| *Δvc0176:KanR* | fwd | TTCCCTGAGTCCAAAAGCGAGG |
| *Δvc0176:KanR* | rev | ATGCTCGATGAGTTTTTCTAATCAGAATTGGACACTTATGGCTCATAATCTTGAAGCTCA |
| *Δvc0176:KanR* | fwd | TGAGCTTCAAGATTATGAGCCATAAGTGTCCAATTCTGATTAGAAAAACTCATCGAGCAT |
| *Δvc0176:KanR* | rev | TGTTTTTTGTTAATTAAACACTAAATATAGTGTCTCTATCTATATCTCCGCCCCGTTCGT |
| *Δvc0176:KanR* | fwd | ACGGGGCGGAGATATAGATAGAGACACTATATTTAGTGTTTAATTAACAAAAAACATCAAC |
| *Δvc0176:KanR* | rev | TGATCGGCCCTTATAGCGATTTTG |
| *Δvc0487:KanR (glmS)* | fwd | GACTGAGTAATAAGTAAACTACTGACCTTTTCATCAATAATG |
| *Δvc0487:KanR (glmS)* | rev | GAATTGGATTCCGGGGATCCGTCGACCATAGTTAAACTCCATATTTTTTATACTGTCGC |
| *Δvc0487:KanR (glmS)* | fwd | GGCGCGACAGTATAAAAAATATGGAGTTTAACTATGGTCGACGGATCCCCGGAATC |
| *Δvc0487:KanR (glmS)* | rev | ATCTGGTAGCCCTTTGATTTATTGAACATTTATGTAGGCTGGAGCTGCTTCAT |
| *Δvc0487:KanR (glmS)* | fwd | CGGAGATGAAGCAGCTCCAGCCTACATAAATGTTCAATAAATCAAAGGGCTACCAGAT |
| *Δvc0487:KanR (glmS)* | rev | CTTTGCGTTCGCTCAGATGTAAAAATTC |
| *Δvc0487:KanR (glmS)* | fwd | CCGAATTCTACCACTGAATGACAGTAATATCAC |
| *Δvc0487:KanR (glmS)* | rev | GGTGAAATCCGGTCAATACGATCTCG |
| *Δvc0647:KanR (pnp)* | fwd | GAAGCAACCGTAGTACGTGATGAGC |
| *Δvc0647:KanR (pnp)* | rev | TCGATGAGTTTTTCTAATCAGAATTGGTCGACCATGAGTATTCCTTATTCTCAGGACAAG |
| *Δvc0647:KanR (pnp)* | fwd | CCTGAGAATAAGGAATACTCATGGTCGACCAATTCTGATTAGAAAAACTCATCGAGCATC |
| *Δvc0647:KanR (pnp)* | rev | GTTTTTGCTCCAGCAACGGAAAATTATATCTCCGCCCCGTTCGTAAG |
| *Δvc0647:KanR (pnp)* | fwd | CTTACGAACGGGGCGGAGATATAATTTTCCGTTGCTGGAGCAAAAAC |
| *Δvc0647:KanR (pnp)* | rev | GCTAAGGTTTACCAAAGGGTTGCTC |
| *Δvc0647:KanR (pnp)* | fwd | GCTGGACTACCTGAAAGGTAAAGATCTG |
| *Δvc0647:KanR (pnp)* | rev | CAAGTTAAACACATCGGGCTGCG |
| *Δvc1836:KanR (tolB)* | fwd | GATGTGGGATCCGAATAGTTACGCA |
| *Δvc1836:KanR (tolB)* | rev | ATTCCGGGGATCCGTCGACCACAAGCCTTCCCTTCCTTATTCAG |
| *Δvc1836:KanR (tolB)* | fwd | CCTGAATAAGGAAGGGAAGGCTTGTGGTCGACGGATCCCCGGAAT |
| *Δvc1836:KanR (tolB)* | rev | CCTTTCTATTAAACAGTTACTTGTTATGTAGGCTGGAGCTGCTTC |
| *Δvc1836:KanR (tolB)* | fwd | GAAGCAGCTCCAGCCTACATAACAAGTAACTGTTTAATAGAAAGGAATAGGAAATGCAACTTAACAAAG |
| *Δvc1836:KanR (tolB)* | rev | CACTTTGGAATGAAACACGATCTCAGC |
| *Δvc1836:KanR (tolB)* | fwd | GGATGGTGACAGTCGTTTATGTGC |
| *Δvc1836:KanR (tolB)* | rev | CTGCTGATTGGTTAGTTTGAGAACCTG |
| *Δvc1904:KanR (lrp)* | fwd | GAAGATTTAGCGGCTTACCCATTAGT |
| *Δvc1904:KanR (lrp)* | rev | CGATGAGTTTTTCTAATCAGAATTGGCATTTTTTATTCCACCTTATTACTTCCTTGCAAAAAAATATACT |
| *Δvc1904:KanR (lrp)* | fwd | GAAGTAATAAGGTGGAATAAAAAATGCCAATTCTGATTAGAAAAACTCATCGAGCAT |
| *Δvc1904:KanR (lrp)* | rev | AAAGATCTCCAACATTCGCTTTAAGTTAATCTCCGCCCCGTTCG |
| *Δvc1904:KanR (lrp)* | fwd | GAAATGGCTTACGAACGGGGCGGAGATTAACTTAAAGCGAATGTTGGAGATCTTTCCAC |
| *Δvc1904:KanR (lrp)* | rev | CAATGCCTTGAGCCTCAAGCTG |
| *Δvc1904:KanR (lrp)* | fwd | GTCCGTGTGAGACAAGCTCC |
| *Δvc1904:KanR (lrp)* | rev | ATTGAGTCGAGAAGAAGGAGCCTC |
| *Δvc2199:KanR (flgC)* | fwd | CGTGTCAATTACTGCCACGGATATT |
| *Δvc2199:KanR (flgC)* | rev | CGCGATACGCAAATCCAGTG |
| *Δvc2199:KanR (flgC)* | fwd | CCACACGAATATTTTTTTTGATTTTTGCACCAA |
| *Δvc2199:KanR (flgC)* | rev | GCTCGATGAGTTTTTCTAATCAGAATTGGCATCTATCTACTCCCCTTTAAGCGACTTGG |
| *Δvc2199:KanR (flgC)* | fwd | CCAAGTCGCTTAAAGGGGAGTAGATAGATGCCAATTCTGATTAGAAAAACTCATCGAGC |
| *Δvc2199:KanR (flgC)* | rev | CCGGCCATACGTTAACTCCTTATCCTTAATCTCCGCCCCGTTCGTAAGCCATTTCCGCTC |
| *Δvc2199:KanR (flgC)* | fwd | GAGCGGAAATGGCTTACGAACGGGGCGGAGATTAAGGATAAGGAGTTAACGTATGGCCGG |
| *Δvc2199:KanR (flgC)* | rev | CCTTAAATTAGCTACTGCGAAGCAGTGA |
| *Δvc2091:KanR (sdhC)* | fwd | GTTCAATAAAAAGCGGATGTTGTGATAGCGG |
| *Δvc2091:KanR (sdhC)* | rev | ATCAGAATTGGATTCCGGGGATCCGTCGACCTTGCTCACTCAGCTCCATTGAGCAT |
| *Δvc2091:KanR (sdhC)* | fwd | ATAAATGCTCAATGGAGCTGAGTGAGCAAGGTCGACGGATCCCCGGAAT |
| *Δvc2091:KanR (sdhC)* | rev | AAGAGACATGCTTTACCATACCAGAACTGTAGGCTGGAGCTGCTTCATCT |
| *Δvc2091:KanR (sdhC)* | fwd | CGGAGATGAAGCAGCTCCAGCCTACAGTTCTGGTATGGTAAAGCATGTCTCTT |
| *Δvc2091:KanR (sdhC)* | rev | CAGTTCGTAGAGATCTTCTACGTAAGTTGCATTG |
| *Δvc2091:KanR (sdhC)* | fwd | CATAGCTCAAATCGTTACGTGGGTAGATAAAC |
| *Δvc2091:KanR (sdhC)* | rev | CACCAGATCCAGTGCATACCATTCC |
| *Δvc2562:KanR (cpdB)* | fwd | GTTAGGACGATTCACATACTGCACAGG |
| *Δvc2562:KanR (cpdB)* | rev | ATTCCGGGGATCCGTCGACCATCACAGACTCCAGAATGAGTGAC |
| *Δvc2562:KanR (cpdB)* | fwd | TGTCACTCATTCTGGAGTCTGTGATGGTCGACGGATCCCCGGAAT |
| *Δvc2562:KanR (cpdB)* | rev | TTTCAATAGGGGCATTATCGGTGCTTATGTAGGCTGGAGCTGCTTC |
| *Δvc2562:KanR (cpdB)* | fwd | GAAGCAGCTCCAGCCTACATAAGCACCGATAATGCCCCTATTG |
| *Δvc2562:KanR (cpdB)* | rev | CAGTTGACGTAAGACTTTGGGCT |
| *Δvc2562:KanR (cpdB)* | fwd | GTCGTAGAGCACCACATCCG |
| *Δvc2562:KanR (cpdB)* | rev | GGTGACCGCTAGAGCGC |
| *lacZ*:p_dns_-*dns*:*lacZ* | fwd | CAAAGCGACATCCTGCTCAATTGC |
| *lacZ*:p_dns_-*dns*:*lacZ* | rev | ATAGAAGTGGTCTCAGCCAATCAAAGGCGTGTGAAAGCGTCATCC |
| *lacZ*:p_dns_-*dns*:*lacZ* | fwd | TGGGGATGACGCTTTCACACGCCTTTGATTGGCTGAGACCACTTC |
| *lacZ*:p_dns_-*dns*:*lacZ* | rev | CTTCTTTACTCCTCGGCTTGAGGGATGTCAGTTCGGGCATTGCTCACG |
| *lacZ*:p_dns_-*dns*:*lacZ* | fwd | TTTGTGCGTGAGCAATGCCCGAACTGACATCCCTCAAGCCGAGG |
| *lacZ*:p_dns_-*dns*:*lacZ* | rev | GAGAGCACAAGGAGGGTGATTG |
| *lacZ check* | fwd | ATGGCAACCATAAAAGACGTAGC |
| *lacZ check* | rev | TTAAGGCTCTCTTTTTTGTGCCAC |
| *vc1807* | fwd | TTTAAAGGGGATCAGTGACCG |
| *vc1807* | rev | CAATTTTGCTTTTGGACCATCCC |
